# Current applications and future direction of MR mammography

**DOI:** 10.1038/sj.bjc.6600713

**Published:** 2003-01-28

**Authors:** P J Kneeshaw, L W Turnbull, P J Drew

**Affiliations:** 1 Academic Surgical Unit, Castle Hill Hospital, Castle Road, Cottingham HU 16 5JQ, UK; 2 The Centre for Magnetic Resonance Investigations, Hull Royal Infirmary, Hull, UK

**Keywords:** magnetic resonance mammography, breast imaging

## Abstract

Compared with triple assessment for symptomatic and occult breast disease, magnetic resonance mammography (MRM) offers higher sensitivity for the detection of multifocal cancer, which is important in selecting patients appropriately for breast-conserving surgery. It is an ideal tool for the screening of patients with a high risk of breast cancer or where there is axillary disease or nipple discharge and conventional imaging has not revealed the primary focus. Techniques are now available to biopsy lesions only apparent on MRM. MRM can differentiate scar tissue from tumour; therefore, it is useful in patients in which there is possible recurrent disease. Clinical and X-ray mammographic assessment of response to neoadjuvant chemotherapy may be unreliable because of replacement of the tumour with scar tissue. MRM can identify responders and nonresponders with more accuracy. It is the modality of choice for the assessment of breast implants for rupture with accuracy higher than X-ray mammography and ultrasound. Advances in both spatial and temporal resolutions, the imaging sequences employed, pharmacokinetic modelling of contrast uptake, the use of dedicated and now phased-array breast coils, and gadolinium-based contrast agents have all played their part in the advancement of this imaging technique. Despite the limitations of patient compliance, scan-time and cost, this review describes how MRM has become a valuable tool in breast disease, especially in cases of diagnostic uncertainty. However, MRM must make the transition from research institutions into routine clinical practice.

The use of magnetic resonance imaging (MRI) in the evaluation of breast disease has continued to gain recognition over the last 15 years, although mainly in research institutions. However, despite its apparent advantages, magnetic resonance mammography (MRM) still awaits introduction into routine clinical practice.

MRM relies on the presence of well-established morphological features that help distinguish malignant from benign lesions. In addition, angiogenesis induced by cancers is demonstrated by dynamic contrast-enhanced magnetic resonance imaging (DCE-MRI). Areas of increased microvessel density are delineated following intravenous gadolinium–diethylenetriaminepentaacetic acid (Gd–DTPA). The enhancement in malignant tissue is thought to be because of increased permeability, vascularity and increased interstitial space (Vaupel, 1994) (Figure 1

**Figure 1:**
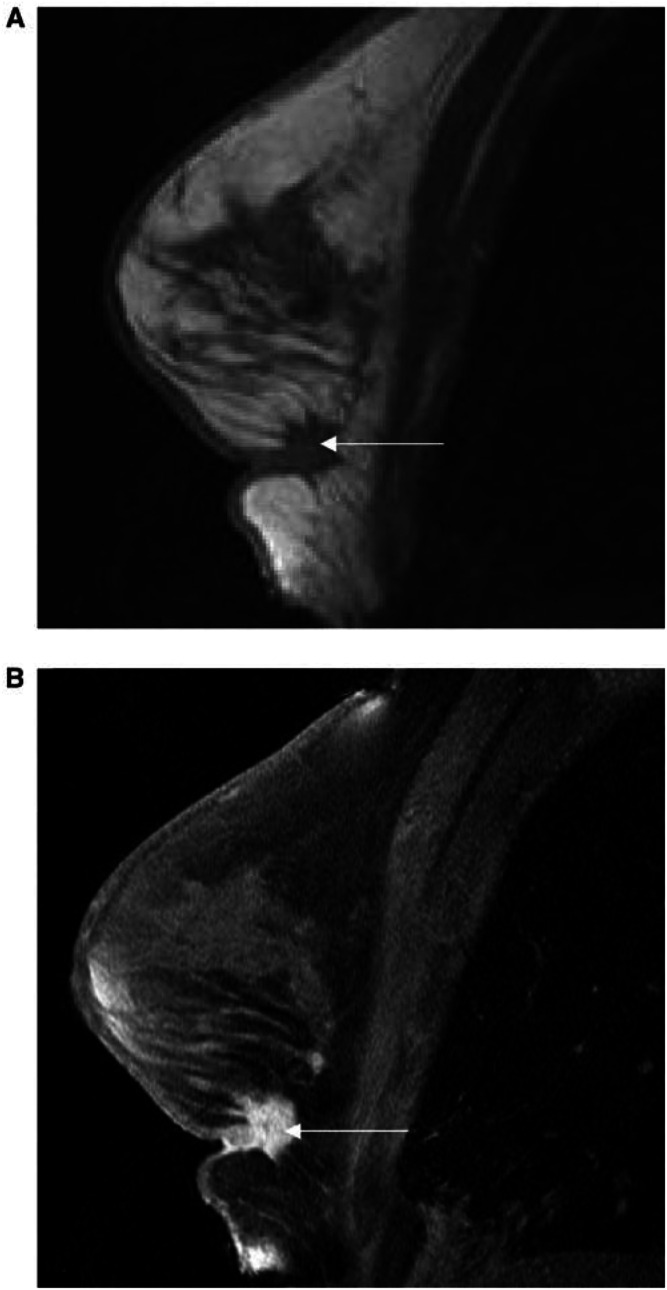
A T1-weighted image of an invasive breast cancer (**A**) fat-saturated image after gadolinium contrast (**B**).

)

In dynamic imaging areas of parenchymal deformity in the breast may be re-imaged over time, thus allowing the signal-intensity – time curve to be analysed in great detail. Fast scanning techniques now make it possible to image preselected areas of the breast at least every 2 s. Curve analysis can be carried out by a number of techniques, most typically by empirical methods or pharmacokinetic modelling.

Kuhl *et al* reported a correlation between the shapes of the signal-intensity curves and the likely aetiology. The curves were subdivided into four categories. Types Ia or Ib are typical of benign lesions, and types II and III are consistent with malignant lesions. Using this method, Kuhl reported a sensitivity of 91%, a specificity of 83% and an accuracy of 86% in distinguishing benign from malignant lesions (Kuhl *et al*, 1999) (Figure 2

**Figure 2:**
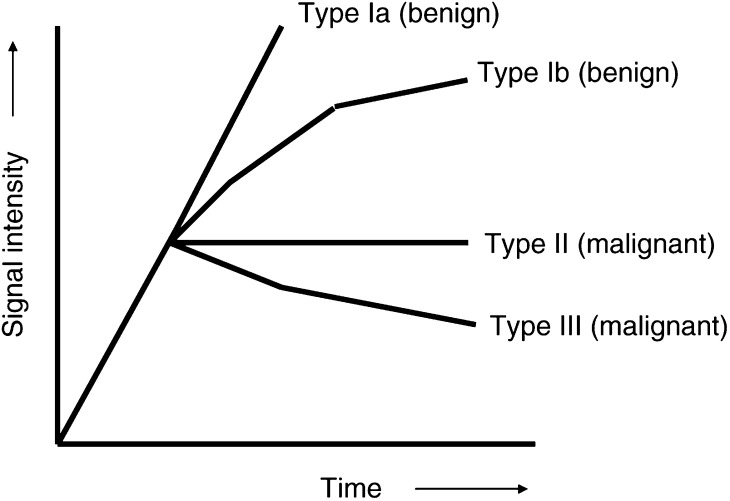
Shapes of signal-intensity curves and likely histology (Kuhl *et al*, 1999).

).

Pharmacokinetic modelling is a mathematical process that uses all the signal intensity data to give numeric values of the permeability and the contrast exchange rate between the plasma and the extravascular and extracellular space of a lesion. These data can be used to differentiate benign from malignant lesions objectively (Buckley *et al*, 1994) (Figure 3

**Figure 3:**
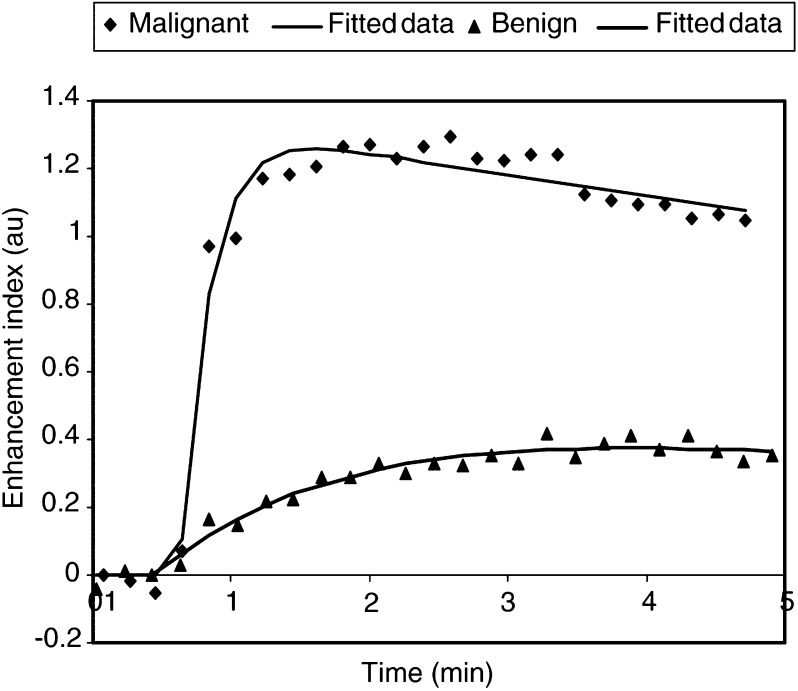
Typical malignant and benign lesion signal-intensity curves (data points) and two-compartment pharmacokinetic modelling (lines).

).

## Symptomatic disease

Currently, triple assessment is the gold standard for symptomatic breast disease. In our centre, we have compared the evaluation of 285 symptomatic patients presenting to the breast clinic by both the triple assessment and MRI. The sensitivity of each modality was as follows: clinical examination 84%, mammography 87.6%, fine-needle aspiration cytology 79.1%, triple assessment 99.2% and MRI 99.2%. In addition, histologically confirmed multifocal disease was detected by MRI in 40 patients, but in only nine patients (22.5%) on mammography. The specificity for the diagnosis of benign disease was as follows: clinical examination 83.1%, ultrasound 88.9%, mammography 86.4%, fine-needle aspiration cytology 97%, triple assessment 59.1% and MRI 90.9%. We concluded that DCE-MRI of the breast is as sensitive and more specific than the combined traditional triple assessment for the diagnosis of malignant breast lesions (Drew *et al*, 1999b). Similar findings have been reported in several other studies ( Table1

**Table 1:** Selected published results of breast MRI

**Author**	**Year**	**Number of lesions**	**Number of cancers**	**Sensitivity**	**Specificity**
Heywang (Heywang-Kobrunner *et al*, 1989)	1989	150	71	0.98	0.65
Kaiser (Buckley *et al*, 1997)	1989	191	58	1.0	0.97
Gilles (Bradley *et al*, 2000)	1994	143	64	0.95	0.53
Orel (Orel *et al*, 1995)	1995	166	64	0.92	0.88
Bone (Brown *et al*, 2000)	1997	238	145	0.92	0.72
Drew (Drew *et al*, 1998)	1998	105	9	1.0	0.93
Lui (Liu *et al*, 1998)	1998	120	70	0.93	0.74
Malich (Malich *et al*, 2001)	2001	90	62	0.98	0.81

).

## Occult disease

The accurate staging of primary breast cancer is paramount. The surgical plan depends upon the size and position of the mass. The presence of multiple tumours in the same quadrant (multifocal) or in multiple quadrants (multicentric) must be determined. Mammography is still the main imaging modality in breast cancer with sensitivity between 69 and 90% and the specificity ranging from 10 to 40% (Rankin, 2000). Tumours may be missed because of observer error, dense breast tissue or even poor technique. Using dynamic MRI of the breast, sensitivities are in excess of 95%, with specificities over 80% (Turnbull, 2000). In a recent study by Fischer *et al*, 16% of candidates for breast-conserving surgery had clinically relevant findings at MR examination (Fischer *et al*, 1999). In a similar but smaller study, Tan *et al* analysed the impact of MRI on the surgical management of 83 patients being considered for breast-conserving surgery. MRI altered the management in 18% of the patients (Tan *et al*, 1999).

We evaluated the accuracy of triple assessment compared with MRI for the detection of multifocal disease prior to breast surgery. The resultant sensitivity, specificity, positive and negative predictive values were 18, 100, 100 and 76% for triple assessment and 100, 86, 73 and 100% for DCE-MRI. The study also identified a subgroup of breast cancer patients with multifocal/multicentric disease not evident on standard triple assessment (Drew *et al*, 1999a).

## Comice trial

The clinical relevance of preoperative detection of small additional carcinomas by MRI, especially small foci of DCIS has yet to be answered. Should these cancers be excised or would adjuvant radiotherapy treat them satisfactorily? The UK-based comparative effectiveness of MR imaging in breast cancer (COMICE) trial has just commenced. This is a randomised control trial recruiting patients planned for wide local excision for primary breast cancer into two study arms. One group of patients will undergo standard imaging and the other group will have additional preoperative MRI staging. This trial will also clarify whether patients scheduled for conservative breast surgery are being adequately staged as the average re-excision rate nationally is currently 14.2% (BASO Specialist Group meeting, 2000).

Screening X-ray mammography has resulted in the detection of increasing numbers of T_1b_ breast lesions, tumour diameter >0.5 cm and ⩽1 cm (Beahrs, 1992). There are little data in the literature regarding the accuracy of diagnosis in sub-T_1b_ breast lesions by any of the routinely used imaging modalities, namely X-ray mammography, ultrasound or MRI. In a recent study of 63 patients, we evaluated the accuracy of diagnosis of clinically occult sub-1 cm lesions using DCE-MRI and achieved a sensitivity of 93.2% and a negative predictive value of 84.6% in differentiating benign from malignant sub-1 cm lesions (Kneeshaw *et al*, 2001a). Therefore, DCE-MRI may have a role following failed stereotactic biopsy or ultrasound-guided core biopsy in patients with very small lesions instead of proceeding to open localised surgical biopsy.

## MR-guided breast biopsy and localisation

The high sensitivity of MRI sometimes means that abnormalities in the breast are detected that cannot be imaged using X-ray mammography or ultrasound. Therefore, MR-guided biopsy techniques are currently being developed. Open configuration scanners allow continuous access to the patient but are limited by low field strength which compromises signal-to-noise ratio (Gould *et al*, 1998). Heywang-Kobrunner *et al* described a unilateral open-breast coil. This allowed successful hook wire placement in 10 out of 11 patients with an average lesion size of 12 mm (Heywang-Kobrunner *et al*, 1994). Closed magnet systems allow access to the patient outside the bore of the magnet with a localisation device. Early small studies using freehand and localisation devices have been encouraging. Daniel *et al* managed to obtain diagnostic tissue in all 27 lesions using a freehand method. Liney *et al* (2000) advocated the use of carbon-fibre-core biopsy needle to reduce artefact. In a larger series of 59 patients with lesions only visible on MRM, Kuhl *et al* (2001) achieved a diagnostic accuracy of 98% using MR-guided stereotactic large-core (14 G) needle biopsy.

## Screening

Breast MRI, with its high sensitivity, is potentially an ideal tool for the screening of high-risk populations. It is especially useful in younger women because of the lower sensitivity of X-ray mammography in this group. Stoutjesdijk *et al* published results in 2001 comparing MRI and mammography in women with a hereditary risk of breast cancer. In a retrospective study of 179 women at high risk of breast cancer, MRI demonstrated 13 cancers, seven of which were not demonstrated by mammography. Receiver operator curves areas for MRI and mammography were 0.99 and 0.74, respectively (Stoutjesdijk *et al*, 2001). Warner *et al* (2002) compared MRI, mammography and ultrasound for surveillance of 196 women with proven BRCA1 or BRCA2 mutations or strong a strong family history of breast cancer. All six invasive breast cancers were picked up by MRI with mammography and ultrasound detecting 2 and 3, respectively (Warner *et al*, 2002).

The UK magnetic resonance imaging for breast screening (MARIBS) trial is a multicentre ongoing trial comparing X-ray mammography and MRI as a method for screening genetically high-risk women. Women aged 35–50 years with BRCA1 or BRCA2 or aged 25–50 years with TP53 are being recruited. Furthermore, this study is examining women's attitudes to MR examination of the breast (Brown *et al*, 2000).

## Response to chemotherapy

**Figure 4:**
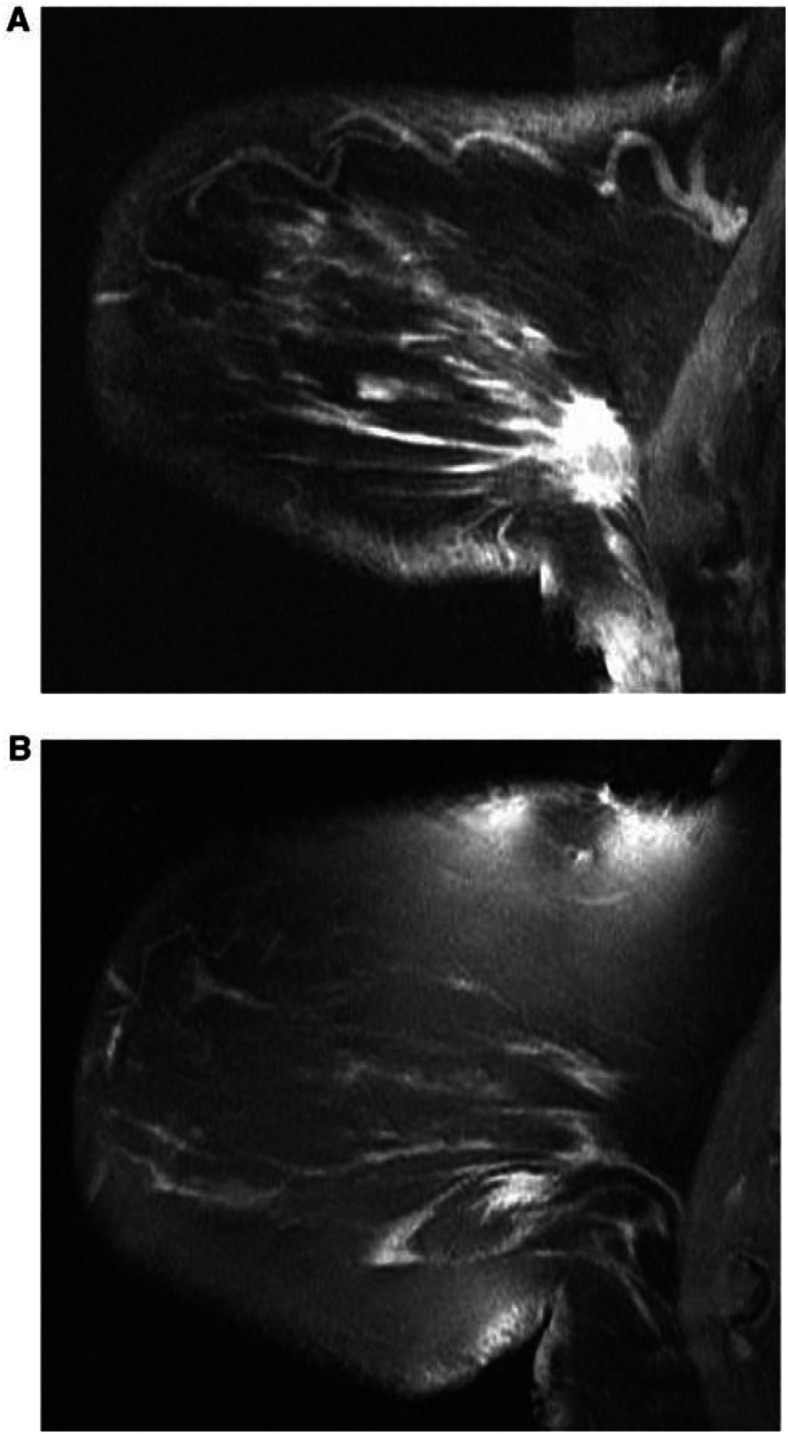
Monitoring the response to neo-adjuvant chemotherapy. Before treatment (**A**) and response after 3 months (**B**).

Patients with locally advanced disease at presentation are more commonly undergoing neoadjuvant chemotherapy followed by surgery. It is the aim of the treatment to shrink the tumour to allow subsequent mastectomy or breast-conserving surgery (Jacquillat *et al*, 1989). Evaluation of the response to chemotherapy by conventional imaging methodology is difficult. This is because of changes induced in the breast parenchyma, including the replacement of tumour with diffuse fibrosis. The fibrotic tissue may be confused with residual tumour on palpation. Breast MRI is more effective than X-ray mammography at determining the extent of residual disease following neoadjuvant chemotherapy (Esserman *et al*, 1999; Drew *et al*, 2001) (Figure 4

).

Tissue permeability may be evaluated using dynamic breast MRI and this can be used to predict patients who will respond to chemotherapy (Hayes *et al*, 2002). Predicting tumour response may allow drugs to be altered early in treatment.

## DCIS

**Figure 5:**
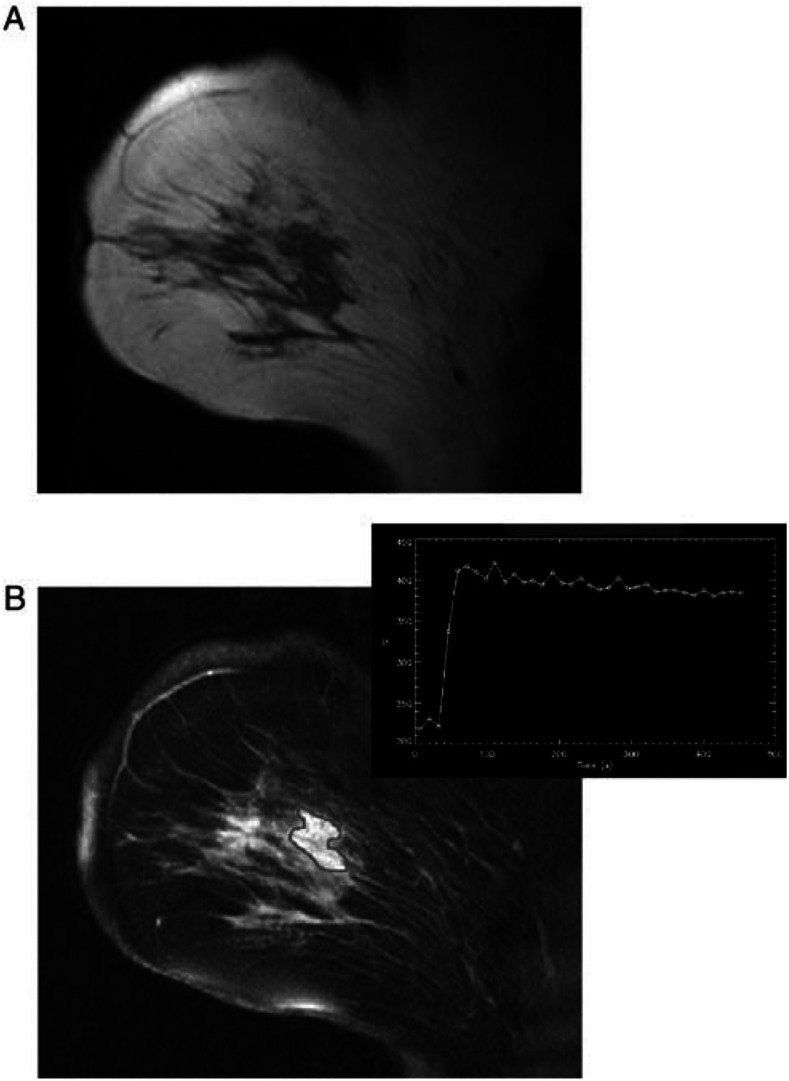
T1-weighted image of DCIS (**A**) and fat-saturated image after gadolinium contrast (**B**) with malignant signal-intensity curve of enclosed area.

The advent of breast screening has shown that *in situ* disease with or without microinvasion represents 20–25% of all screen-detected breast cancer (Roberts *et al*, 1990). The MR appearance of microcalcification, the cardinal mammographic feature of DCIS is a signal void. However, in a similar manner to invasive disease, DCIS induces angiogenesis and this can be detected *in vivo* by DCE-MRI (Figure 5

). In a study by Gilles *et al* (1995), dynamic MRI showed contrast enhancement in 34 out of 36 patients with DCIS. Other MR studies have reported variable accuracy for classification of microcalcification. Westerhof *et al* (1998), investigating mammographically suspicious microcalcifications, reported a sensitivity of 45%, specificity of 72%, positive predictive value of 71%, negative predictive value 46% and an accuracy of 56%, and in a further study, Gilles *et al* observed a sensitivity of 95% and a specificity of 51%. Their specificity was impaired because the presence or absence of contrast uptake in the breast was the only parameter used to decide if the area of microcalcification was associated with malignancy or not (Gilles *et al*, 1996).

Malich *et al* looked specifically at 100 mammographically suspicious lesions using different imaging modalities. He showed MRI to have a sensitivity of 98%, specificity of 81%, positive predictive value of 88% and negative predictive value of 97% for malignant disease, while the corresponding values for DCIS and hyperplasia were 86, 83, 75 and 91%, respectively (Malich *et al*, 2001).

We are currently evaluating the use of DCE-MRI in the assessment of screening-detected mammographically indeterminate microcalcifications and initial results for differentiating benign from malignant causes are encouraging. In a cohort of 42 patients, we achieved a sensitivity of 87.5% and a negative predictive value of 80% (Kneeshaw *et al*, 2001b).

## Recurrent disease

Areas of scar tissue following breast-conserving surgery can simulate recurrences on mammography. MRI performed up to 1-year post-surgery shows some enhancement in the scar tissues at dynamic examination making the differentiation of scar from recurrence unsatisfactory. After 12 months, however, MRI can be used to detect or exclude recurrent tumour with high sensitivity. Kramer *et al* (1998) reported a sensitivity of 91% for detection of cancer recurrence with MRI compared to 51, 67 and 83% for physical examination, X-ray mammography and ultrasound, respectively, in 33 cases of tumour recurrence. We evaluated clinical examination, mammography and MRI in the detection of recurrent tumour following breast-conserving surgery. The sensitivity of clinical examination, mammography, examination combined with mammography, and MRI alone for the detection of recurrent cancer were 89, 67, 100 and 100%, respectively, with specificity values of 76, 85, 67 and 93%. We concluded that combined clinical examination and mammography were as sensitive as dedicated dynamic MR of the breast for the detection of locoregional recurrence, but that breast MRI was associated with a far greater specificity (Drew *et al*, 1998).

This year Belli *et al* evaluated 40 women with clinical or radiological evidence of local recurrence (Belli *et al*, 2002). MRI identified all the 22 cancers and showed 95% accuracy, 100% sensitivity, 88.8% specificity with 5% false positives and 100% negative predictive values.

There are no studies to date evaluating the use of MR for monitoring patients after breast-conserving surgery. However, the Board of the Faculty of Clinical Radiology from The Royal College of Radiologists in their Guidance on Screening and Symptomatic Breast Imaging published 1999 stated that where conventional triple assessment has been unhelpful in the assessment of patients with suspected recurrences, MRI has been shown to be useful.

## Axillary disease

Breast cancer may present as an axillary metastasis with normal triple assessment. MRI has been used in this setting to detect the primary cancer. In one such study Orel *et al* (2000b) demonstrated the primary cancer in 19 out of 22 patients who presented with an axillary metastasis.

Clinical examination is generally poor in evaluating the axilla for recurrent tumour. Bradley *et al* evaluated the accuracy of axillary MRI in distinguishing between recurrent tumour and treatment-induced effects. In their series using MRI, the specificity and sensitivity for the detection of axillary metastasis was 100 and 89%, respectively (Bradley *et al*, 2000).

## Nipple discharge

Nipple discharge, although usually benign in origin, causes concern because of the possibility of an underlying cancer. Worrying features such as spontaneous, unilateral discharge in which the fluid is bloody, serous or clear warrants further evaluation. In a study by Orel *et al* (2000a), the use of MRI in the investigation of nipple discharge was evaluated. Mammographic findings proved negative in 22 out of 23 patients while in 73% of the patients, MR findings correlated with the pathology findings. They concluded that MR imaging could help identify both malignant and benign causes of nipple discharge (Orel *et al*, 2000a).

## Breast implants

**Figure 6:**
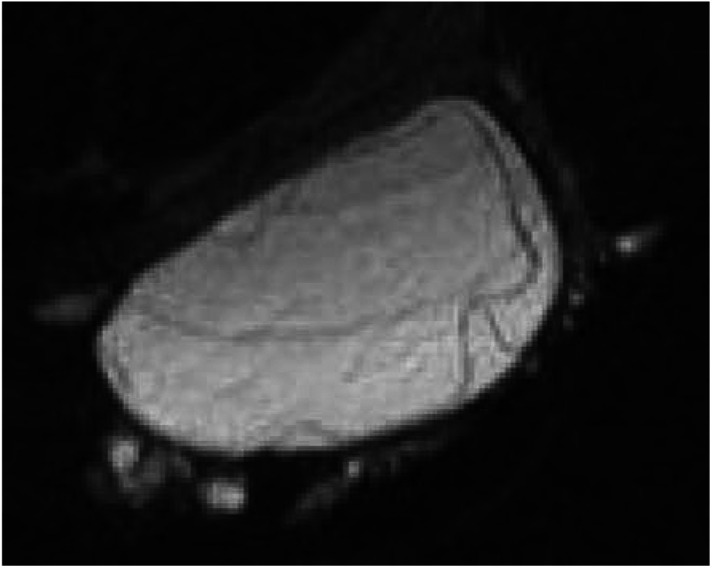
Silicone-specific series showing an extracapsular tear with silicone globules in soft tissues.

MRI is widely regarded as the investigation of choice for the demonstration of intracapsular and extracapsular breast implant ruptures. The sensitivity of MR for the detection of implant rupture is around 95% with a specificity of 90% and is considerably better than mammography or ultrasound (Turnbull, 2000). The intracapsular rupture is recognised by the presence of the *linguine sign*, which is caused by extensive folding of the collapsed shell of the implant (Gorczyca *et al*, 1994). Free silicone may be seen in the parenchyma of the breast following an extracapsular tear with silicone-specific MRI sequences (Figure 6

).

## Practical limitations

The patient is required to remain still within the scanner for around 20 min for a standard dynamic MR series. Patients who are large or have other medical problems may not tolerate these conditions reducing the quality of the examination. Careful and considerate patient positioning may help to overcome these problems.

Patients with claustrophobia may initially refuse to enter the scanner. Careful explanation about the examination often helps to overcome these problems. Breast scanning is generally performed with the patient prone and the breasts positioned in the cups of the dedicated breast coil. This position is often well tolerated by anxious patients, as they can see out of the magnet, but in severe cases a light sedative may be administered. However, in our centre, sedation has never been required for a breast MR examination because of the favourable patient positioning. If one examines MRI of all body parts, the sedation rate is approximately 4%.

Gadolinium contrast must be given intravenously during the examination. Previous allergy to Gd–DTPA is a contraindication; however, true anaphylactic shock following gadolinium administration occurs in only 0.0003% of patients (Niendorf *et al*, 1998).

Previous surgery, especially cardiac, may cause image distortion because of metal artefact from valves or sternal closure wire.

Absolute contraindications to MRI are mainly because of the presence of ferromagnetic materials. Any non-MRI compliant implant needs to be investigated for MRI compatibility. Any suspicion of a metal injury including foreign bodies in the eye or injuries from shrapnel needs to be excluded.

Following the initial financial outlay for an MR scanner, the running costs are still significant with an average scan costing around £350 including contrast agents.

## Specificity issues

MRI of the premenopausal breast may suffer reduced specificity because of the presence of ‘hormone reactive’ parts of the breast. These ‘pseudo-lesions’ enhance following Gd–DTPA variably throughout the menstrual cycle. Kuhl *et al* (1997) reported that the least troublesome time for scanning is within week 2 of the menstrual cycle. The use of hormone replacement therapy will also alter enhancement of the breast (Reichenbach *et al*, 1999).

Vascular fibroadenomas and papillomas form a large proportion of false positives as they can enhance in a similar way to malignant lesions. The commercially available Gd-chelates are extracellular, nontissue-specific compounds that leak out of the intravascular space. New macromolecular contrast agents leak through cancer microvessels, but do not leak through the endothelial barrier of benign tumours (Gerlowski and Jain, 1986). These blood-pool contrast agents are currently being developed. These may provide a marked improvement in the ability to examine the macrocirculation as well as microcirculation and thereby increase specificity.

Although DCE-MRI has been shown to be effective in distinguishing scar from recurrent tumour following breast surgery, it can be limited when used immediately following surgery. Interpretation of images may be compromised because of haemorrhage.

**Figure 7:**
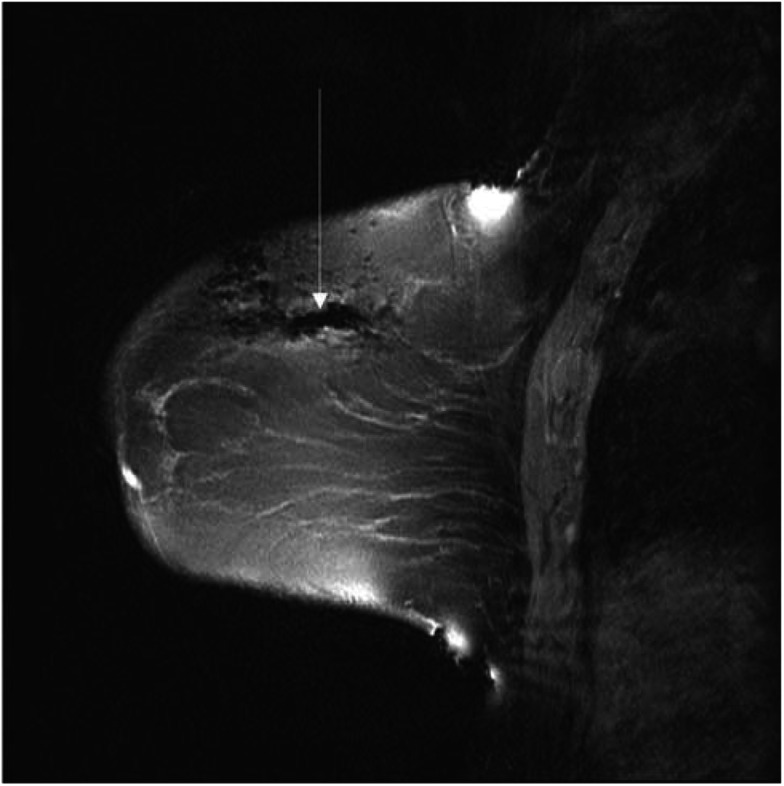
T1-weighted postcontrast fat-suppressed image of a breast showing signal voids from surgical instrument metal fragments.

The presence of metal clips or even tiny amounts of metal rubbed off surgical instruments can severely reduce the image quality caused by metal artefact (Figure 7

).

## Future

The progression of MR technology will uncover smaller lesions that are occult on X-ray mammography, ultrasound and clinical evaluation. 3T systems with multichannel receivers will undoubtedly increase both spatial and temporal resolutions and the use of 4D sequences will provide full breast coverage. Using special pulse sequences and a thin slice thickness, small lesions will be evaluated using both contrast uptake and morphologic parameters. The development of tumour-specific contrast agents may provide a simple answer to the issues of specificity. If the nature of these lesions cannot be clarified using noninvasive MR methods, then the development of MR-guided biopsy or MR-guided surgery will have to continue. However, further trials are needed as to the clinical relevance of these very small invasive or *in situ* carcinomas especially when these are multifocal.

MRI of the breast has shown great potential in the evaluation of breast disease in research centres; however, its acceptance into common practice is limited. This may be because of the availability of MR systems with long waiting times in many centres. This may improve with the introduction of additional new opportunities funding (NOF) systems. There is also a national shortage of radiologists, especially those who specialise in MR and breast disease. Advances in software will enable compression of the huge MR data sets, particularly dynamic contrast-enhanced data with functional images corresponding to contrast uptake characteristics. This may help to reduce the time taken to report MR breast examinations.

The variety of scanning techniques and hardware limit reproducible results from centre to centre. To gain widespread usage, we must develop systems that exceed current standards of imaging but will not require complex data analysis. We must standardise protocols compatible with the hardware that is available to the majority of hospitals. In the meantime, breast MRI will continue to be regarded as a problem-solving tool.
